# Advances of Stimulus-Responsive Hydrogels for Bone Defects Repair in Tissue Engineering

**DOI:** 10.3390/gels8060389

**Published:** 2022-06-20

**Authors:** Shuai Chang, Shaobo Wang, Zhongjun Liu, Xing Wang

**Affiliations:** 1 Department of Orthopedics, Peking University Third Hospital, Beijing 100191, China; changshuaipkuth@163.com (S.C.); wangshaobopkuth@163.com (S.W.); 2 Beijing Key Laboratory of Spinal Disease Research, Peking University Third Hospital, Beijing 100191, China; 3 Engineering Research Center of Bone and Joint Precision Medicine, Ministry of Education, Peking University Third Hospital, Beijing 100191, China; 4 Beijing National Laboratory for Molecular Sciences, Institute of Chemistry, Chinese Academy of Sciences, Beijing 100190, China; 5 Beijing National Laboratory for Molecular Sciences, Institute of Chemistry, University of Chinese Academy of Sciences, Beijing 100049, China

**Keywords:** stimulus-responsive, smart hydrogels, bone defects repair, tissue engineering

## Abstract

Bone defects, as one of the most urgent problems in the orthopedic clinic, have attracted much attention from the biomedical community and society. Hydrogels have been widely used in the biomedical field for tissue engineering research because of their excellent hydrophilicity, biocompatibility, and degradability. Stimulus-responsive hydrogels, as a new type of smart biomaterial, have more advantages in sensing external physical (light, temperature, pressure, electric field, magnetic field, etc.), chemical (pH, redox reaction, ions, etc.), biochemical (glucose, enzymes, etc.) and other different stimuli. They can respond to stimuli such as the characteristics of the 3D shape and solid–liquid phase state, and exhibit special properties (injection ability, self-repair, shape memory, etc.), thus becoming an ideal material to provide cell adhesion, proliferation, and differentiation, and achieve precise bone defect repair. This review is focused on the classification, design concepts, and research progress of stimulus-responsive hydrogels based on different types of external environmental stimuli, aiming at introducing new ideas and methods for repairing complex bone defects.

## 1. Introduction

Bone defects, caused by trauma, bone tumor resection, infection (such as osteomyelitis), and other factors, have become one of the major issues seriously affecting patients’ limb function, causing physiological and psychological damage, and always requiring a safe and effective treatment to achieve bone tissue regeneration and repair. Among previous common treatments, bone transplantation has been the gold standard for the treatment of bone defects [1]. However, bone transplantation has failed to be widely used, due to the limited bone graft source of autologous material, high treatment cost, immune rejection risk, complicate bone handling technology, as well as the risk of infection and complications [2,3]. Therefore, the traditional bone defect treatment technology is unable to effectively meet the needs of patients. Tissue engineering is a multifaceted field that uses biological substitutes to promote the regeneration of failing or damaged tissues. The basic element of tissue engineering relies on cells, scaffolds, and growth factors. Traditional strategies in tissue engineering always incorporate the use of stem cells to regenerate new tissue in damaged areas [4,5,6,7,8]. In this case, various scaffolds provide attachment sites for cells and a protective environment for the proliferation and differentiation of attached cells. To successfully achieve bone tissue regeneration, it is important to find a scaffold that can temporarily replace the damaged tissue and adapt to the biological environment of the host tissue with optimal porosity, thus allowing for the transport of a sufficient number of cells. In addition, scaffold must biodegrade within the host tissue at an appropriate rate. The proposed biomaterial must be able to interact with the surrounding tissue, and the patient’s immune response must be minimized. Furthermore, the scaffold should provide adequate mechanical strength to the bone defect site and efficiently encapsulate and control the release of drugs or proteins [9,10].

It is well known that the process of bone defect repair is composed of an organic range of osteocytes, osteoblasts, and osteoclasts, called basic multicellular units (BMUS). BMUS act on the periosteum, trabecular surface, and cortex of bone, promoting osteogenesis absorption and bone repair. However, the adhesion, proliferation, and differentiation of BMUS should have a suitable cellular microenvironment and extracellular matrix (ECM), and, thereby, the question of how to simulate the natural ECM becomes the key to tissue engineering scaffolds [11]. The ECM is composed of a mixture of several biomolecules, including collagens and glycoproteins, arranged in distinct structures that are essentially unique to a specific tissue. The bone ECM determines the mechanical properties of the skeleton. The mineralized portion of the bone tissue imparts rigidity to the biomaterial, while the organic components of the ECM provide flexibility. The mineralized portion of the ECM is composed largely of calcium phosphate in the form of hydroxyapatite (HAP), plus an extensive type I collagen-rich organic ECM. In addition to mineralized bone ECM, other unique tissue types exist in association with bone ECM networks, such as non-mineralized marrow, the endosteum, the periosteum, and the perilucanar matrix, which can regulate the processes of osteoclast activity, osteoblast progenitor proliferation and differentiation, and osteocyte function. Until now, ECM has played a far more dynamic role in regulating cell function, tissue morphogenesis, modulating the matrix assembly, and organizing the process of matrix mineralization for bone remodeling. In addition, the ECM can bind to extracellular growth factors, cell-bound ligands and receptors, and proteases to exert its activities and functions [12,13,14,15,16].

Hydrogels have a hydrophilic nature with a three-dimensional structure and similar ECM components, which are easily chemically modifiable and can be further tuned to exhibit a favorable degradation profile and mechanical integrity, as well as incorporating cells, growth factors, or drugs, making them suitable scaffolds for cellular infiltration, adhesion, growth, proliferation, migration, and differentiation [17,18,19]. Moreover, the sustainable development of dynamic chemistries allows the use of hydrogels as nearly physiological matrices to recapitulate the dynamic interactions of native environments [20,21,22]. Therefore, based on the excellent hydrophilicity, adjustability, biocompatibility, and degradability for cell adhesion, proliferation, and differentiation, hydrogel scaffolds have been widely used in the biomedical field of tissue engineering research [23]. According to the differences in the source of gel materials, hydrogels are divided into synthetic polymer hydrogels and natural polymer hydrogels. On the one hand, the traditional polymer hydrogel mostly contains synthetic polymer materials, such as polyethylene glycol (PEG), polyacrylic acid (PAA), polymethyl methacrylate (PMMA), and so on. In addition, there is a complex synthesis process, large energy consumption, high cost, degradation in the rate of bone regeneration, and the elastic modulus cannot adapt to the needs of the specific microtissue environment, which has been an important reason restricting their application [24,25,26,27,28,29,30]. In addition, the performance of traditional hydrogel molding is individual and unchangeable in the treatment of large and complex bone defects, and it cannot accurately fill irregular defect parts. The above defects greatly limit its application breadth in bone defect repair. On the other hand, since natural materials can be used to conduct multiple structural and biological functions due to their outstanding range of macromolecular designs, which have originated from the evolution of living beings in different environments throughout millions of years, naturally derived hydrogels (chitosan, alginate, hyaluronan, collagen, agarose, etc.) are particularly appealing because of their inherent biocompatibility, biodegradability, safety, and accessible renewable resources, such as animals, plants, algae, and microorganisms around the world [31,32,33]. After the complex purification, fermentation, and other steps, these natural polymer hydrogels can achieve large-scale production to maintain the original biocompatibility and excellent biodegradation performance for a wide range of applications [34,35,36].

**Figure 1:**
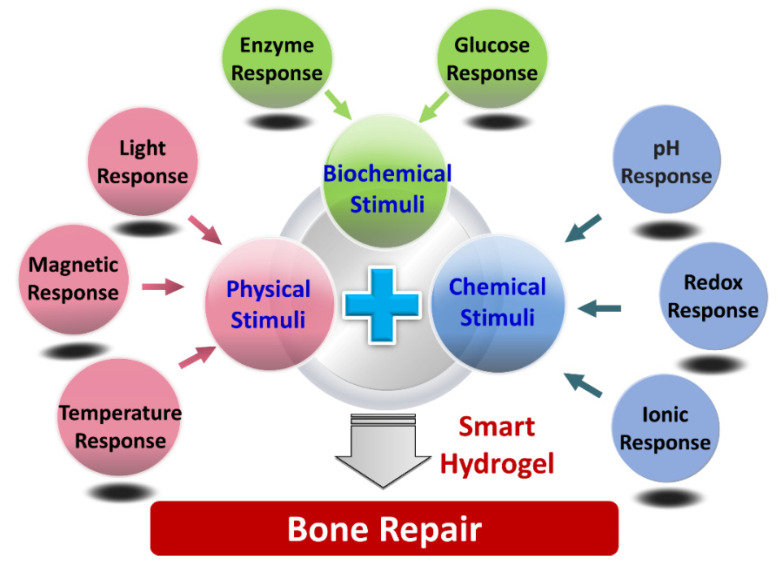
Schematic illustration of different types of smart hydrogels for bone repair.

According to the types of stimulus sources, hydrogels are mainly divided into three categories (Figure 1): physical responsive hydrogels, chemical responsive hydrogels, and biochemical responsive hydrogels [37]. Since 1960, when Wichterle published the landmark article [38], the hydrogel has gradually developed from a simple, inert gel material to a complex, variable and controllable “smart” gel material with sensitive or responsive properties, which has been gradually introduced into the field of bone defect repair. It can respond to external physical stimuli (light, temperature, pressure, electric field, magnetic field, etc.), chemical stimuli (pH, redox response, ions, etc.), and biochemical stimuli, glucose, enzymes, etc.), and conduct shape transformation, produce injectability, and exhibit self-healing and shape memory properties to perform complex bone defect repair treatment that is minimally invasive; thus, it has become an emerging scaffold material in bone tissue engineering [39].

Stimulus-responsive hydrogels can be prepared by the design of polymer molecular chains. Altering external environmental stimulation, such as temperature, pH, ionic strength, organic compound concentration, magnetic fields, electric fields, and light, can regulate the structure and physicochemical properties of the hydrogels, thus changing the swelling and degradation behavior of the hydrogels, which endows them with “smart” characteristics [40,41]. This allows smart hydrogels to have environmentally responsive gelation and degradation properties, so stimulus-responsive hydrogels can be widely used for bone tissue engineering. Through molecular design, the reactive hydrogel scaffolds, cells, and growth factors are injected into specific defect tissues, and, meanwhile, the stimulus-responsive hydrogels can not only positively provide mechanical and biological support for bone regeneration, but also gradually be degraded and absorbed by the tissue after implantation into the organism. With the disintegration of the hydrogel scaffold, the cells can constantly proliferate, differentiate, and secrete the ECM, which eventually enables new tissue formation and then achieves the corresponding functional bone reconstruction [42,43].

In this review, we will first introduce the classification and design concept of smart hydrogels based on different types of external environmental stimuli, and then discuss relevant examples of research progress using these stimulus-responsive hydrogels, especially focusing, but not limited to, bone repair applications.

## 2. Stimulus-Responsive Hydrogels

External environmental stimulation mainly includes light, temperature, pH value, redox, ionic strength, magnetic fields, electric fields, glucose, and enzymes. Therefore, the stimulus-responsive hydrogels can be divided into photo-responsive hydrogels, temperature-responsive hydrogels, pH-responsive hydrogels, redox-responsive hydrogels, magnetic-responsive hydrogels, enzyme-responsive hydrogels, and so on [44]. Different external stimulus environments require the corresponding design and fabrication of the hydrogel in the molecular structure. Once these external factors change to a critical point, stimulus-responsive hydrogels can rapidly undergo a discontinuous change or volume phase transformation. They have high biocompatibility, flexible design, high stability, and few side effects, and they can not only transport drugs for targeted release to achieve the purpose of targeted therapy in the field of clinical treatment, but also embed cells and serve as scaffolds for bone tissue repair in regenerative medicine. Table 1 summarizes the advantages and limitations of six responsive hydrogel categories.

### 2.1. Photo-Responsive Hydrogels

Light, as a remote stimulus, can present precise control in space and time [45]. Photo-responsive hydrogels include a polymer network and photochromic groups. These groups undergo light fracture, isomerization, and light dimer formation under different light conditions [46]. Photo-responsive hydrogels can be divided into two categories: covalent connection with the hydrogel (nitrobenzyl) and non-covalently connected but suspended in a network (Irgacure 2959, phenyl-2,4,6-lithium, eosin Y, etc.) of nitrobenzyl with the hydrogel [47,48,49,50,51]. For the first class of hydrogels, the light signal is captured by the photochromic molecules and then converted into a chemical signal by chromophores [52]. The size and mechanics of a photosensitive hydrogel can change due to the radiation of light-sensitive groups, which undergo light fracture, isomerization, and light dimer formation under different light conditions [53], such as crosslinking linearly functionalized polyacrylamide (PA) with a photosensitizer of azobenzene (AZO). For example, the photoinduced AZO-PA hydrogel prepared by Lee et al., changed its stiffness with the visible light wavelength, its degradation rate could match the bone generation time, and its biocompatibility was verified by mesenchymal stem cells. Time-resolved analysis of cell morphology showed characteristic cell spreading and increased aspect ratios in response to greater substrate stiffness. This hydrogel provided a platform to study mechanosignaling in cells responding to dynamic changes in stiffness, which is expected to play an important role in bone defects at different locations, offering a new way to study mechanotransduction signaling pathways and biological processes for bone tissue engineering [54]. For the second class of hydrogels, photoinitiators could decompose into free radicals and undergo a chemical reaction of polymerization or isomerization through the effect of light, causing changes in macromolecular chain conformation and the swelling volume. Khetan et al., reported a method for the gradual preparation of a methacrylic acid hyaluronic acid hydrogel, achieving the initial gelation of the hydrogel by introducing dithiothreitol into the methacrylic acid hyaluronic acid solution. When the initialized hydrogel was swollen by the photoinitiator of Irgacure 2959, the hydrogel could rapidly stiffen due to the free radical polymerization of the remaining methacrylate group [48]. Thus, both types of photoreactive hydrogels could regulate the physical or chemical properties of the hydrogels. The photoreaction fraction was sensitive to specific wavelengths (e.g., visible, ultraviolet, infrared light), which improved the control of the photo-responsive hydrogels [55].

### 2.2. Temperature-Responsive Hydrogels

Temperature-responsive hydrogels or temperature-sensitive hydrogels, based on the temperature difference from room temperature, can produce physical and chemical changes from sol to gel. Temperature-sensitive hydrogels have both hydrophilic and hydrophobic groups and phase transition properties with a temperature response at a critical solution temperature (CST), which could produce a change in affinity to the solvent. When performing the swelling–contraction state transition—that is, the sol–gel state transition—the temperature transition point is called the lowest critical dissolution temperature (LCST) or the utmost critical dissolution temperature (UCST) [56]. The most common LCST temperature-sensitive hydrogel used in bone repair is poly N-isopropyl acrylamide (PNIPAAm) and its derivatives. As a raw material for temperature-sensitive nanogels, by adding more hydrophilic acrylamide (AAm) to NIPAAm, the LCST rises from 32 °C to 37 °C, which is closer to the human body temperature. Yoshimatsu et al., synthesized poly (NIPAAm-co-AAm) copolymers and prepared their nanogels by radical polymerization [57]. The nanogels had a diameter of 50 to 450 nm and a volume phase transition temperature of 37 °C to 43 °C. Animal thermal targeting experiments combined with near-infrared fluorophore showed that gel delivery could be performed at specific locations by controlling the LCST and tissue heating processes of the nanogels. Antitumor drugs can also be loaded onto temperature-responsive nanogels. In bone defects, this nanogel can be targeted to treat cancer and bone defects. The temperature-responsive hydrogel system has been applied to release diverse growth factors and thus serve as a form of bone-regenerative medicine. The growth factors covalently bind to the injectable hydrogels and influence the growth of specific types of cells, which promotes cell proliferation, migration, recruitment, and angiogenesis, and modulates cell differentiation. Nafee et al., reported a temperature-responsive chitosan/β-GP hydrogel to effectively deliver bone resorption inhibitors and hinder the osteoclast action to treat Paget’s disease and postmenopausal and glucocorticoid-induced osteoporosis. After encapsulating a BCS III bone resorption inhibitor, alendronate (ALN), the chitosan/β-GP hydrogel exhibited temperature-reversible gelation behavior and ensured controlled ALN release over 45–65 days, with a lower inflammatory response and faster proliferation and maturation of the granulation tissue. The biodegradability and biocompatibility of the system were confirmed upon analysis at 21 days after injection of the hydrogel [58].

**Figure 2:**
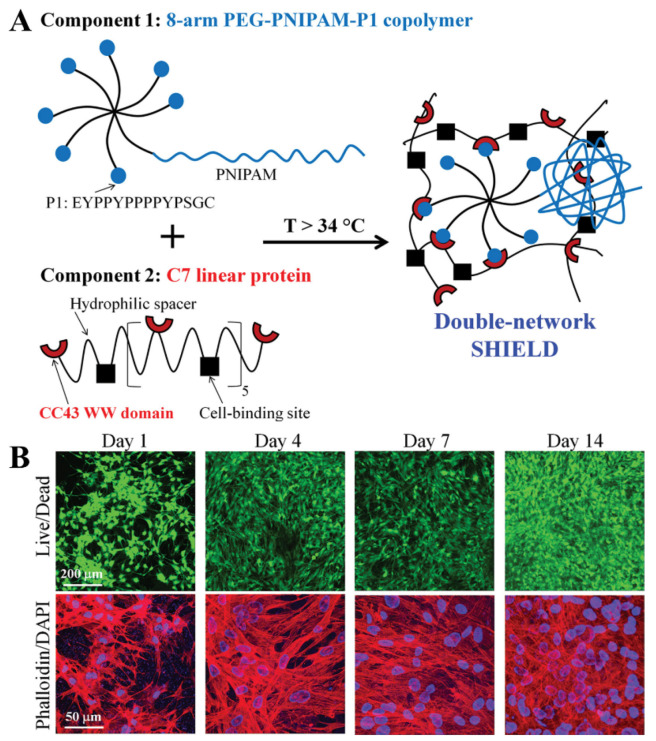
Retention of transplanted stem cells enhanced by thermo-responsive injectable hydrogels with in situ double network formation. (**A**) Structural design of SHIELD. (**B**) Confocal images of hASCs cultured in SHIELD. Reproduced from [61] with permission. Copyright 2015 Wiley.

In addition, copolymers of PEG and polycaprolactone (PCL) are also common LCST-type hydrogels. Ni et al., studied an injectable PEG-PCL-PEG hydrogel as a thermal inducing material for bone tissue engineering with reversibility recovery when PEG-PCL-PEG aqueous solution was changed from sol to gel [59]. This hydrogel had the advantages of being minimally invasive and precision matching in the treatment of bone tissue defects. Fu et al., combined collagen and HAP into a PEG-PCL-PEG copolymer to produce a hydrogel, and its biocompatibility and enhanced biomimetic microstructure endowed it with excellent performance in the treatment of bone defects [60]. Cai et al., prepared a dual network hydrogel (SHIELD) (Figure 2A), which can be used to directly inject transplanted stem cells, such as human adipose stem cells (hASCs) and human bone marrow mesenchymal stem cells (BM-MSCs) [61]. Molecular identification between peptides could form a weak network, which provided mechanical protection during injection and reduced cell damage caused by shear stress during injection. When the temperature increased to SHIELD LCST (ca. 34 ℃), the enhanced hydrophobic interaction between PNIPAm polymer chains strengthened the network structure and improved the cell retention time, thus providing a microenvironment for cell proliferation and differentiation for bone repair (Figure 2B). Traditional temperature-sensitive materials include polyacrylic acid, gelatin, etc. However, their applications in bone repair are scarce because of their high solid-state temperature, which can easily cause damage to the body and is not conducive to material implantation and bone formation.

### 2.3. pH-Responsive Hydrogels

pH is the most intensively studied environmental stimulus in chemical stimulus-responsive hydrogels. The polymer hydrogels have many acidic or alkaline groups, which can quickly receive or release protons (protonation and deprotonation) in the environment, thus enabling an intelligent response to pH solutions. A hydrogel crosslinked by cellulose precursor macromolecules under the catalysis of phenylalanine had a pH response, and the gel time and mechanical strength could be adjusted by the content of precursor macromolecules and phenylalanine [62].

The pH-sensitive hydrogels are generally composed of the polymer backbone and ion side groups, which perform the conversion through the absorption or release of protons in response to the changes in the surrounding pH [63]. When the pH of the surrounding environment reaches pKa or pKb, the ionic strength of pH-sensitive hydrogels changes significantly, resulting in strong electrostatic repulsion and the appearance of ionic groups and volume mutation. Based on the charge properties of the charged groups, the pH-responsive hydrogels are divided into two categories, anionic and cationic hydrogels. Anionic hydrogels with negatively charged groups include carboxylic acid, sulfonic acid, etc. [64]. In high-pH conditions (pH > pKa), the charged groups expand the hydrogel. Similarly, a cationic hydrogel changes in low-pH conditions (pH > pKb). At the same time, some scholars have pointed out that in order to simulate the natural ECM, a weak polymeric electrolyte should be used to synthesize the pH-sensitive hydrogels. The ionic strength in a weakly polymerized electrolyte can be adjusted smoothly by changing the pH solution to achieve the desired mechanical properties [65]. The pH-responsive hydrogels had controllable pH by introducing weak electrolytes at their hydrophobic ends [66]. Yoshikawa et al., synthesized triblock copolymers using pH-sensitive poly(2-(diisopropylamino)ethyl methacrylate) (PDPA) and poly(2-(methacryloyloxy)ethyl phosphorylcholine) (PMPC) [67]. Simple modulation of the solution pH in a narrow physiologically relevant range produced highly adjustable hydrogels with Young’s moduli of 1.4 to 40 kPa. Such hydrogels can simulate the natural environment well, even under complex stresses, and have wide applications in dealing with complex stresses caused by various bone defects. Rogina et al., demonstrated a pH-responsive chitosan–HAP hydrogel with a gelling agent of NaHCO_3_. Tailoring of NaHCO_3_ could achieve the quick gelation of the chitosan–HAP-based hydrogel within 4 min, exhibiting good viability for cell proliferation and differentiation as a potential cell carrier [68]. Lundberg et al., prepared a pH-dependent hydrogel by producing synthetic thiol-functionalized histamine [69]. The hardness of the hydrogels increased five-fold in the range of pH 5.0 to 8.0, and their biocompatibility in different cell lines greatly improved the mechanical properties of the hydrogel scaffolds, indicating promising applications in the repair of large-segment bone defects. 

### 2.4. Redox-Responsive Hydrogels

Redox-responsive hydrogels can react to the reduction and oxidation of their constituent molecules. The redox-responsive hydrogels undergo redox reactions through partial subunits within the polymer backbone, flooding opposing ions to balance the newly formed charges and eventually leading to the material’s expansion [70]. Different metal ions confer different redox responsiveness, resulting in different mechanical properties. Iron ions are considered promising crosslinkers in biomedical applications, providing hydrogels with redox responsiveness and tunable mechanical properties by switching between two oxidation states, trivalent and divalent iron ions. This compensates for the lack of mechanical properties of simple hydrocoagulation and support. Papanikolaou et al., designed a new redox hydrogel that switched between divalent and trivalent iron ions and was reversible between soft (0.06 MPa) and hard (2.1 MPa) [71]. Many scholars have studied the relationship between metal ions and the mechanical properties of materials, and they have also confirmed that the regulation of hydrogel hardness by a redox reaction can promote bone regeneration [72,73,74]. However, further research is needed to investigate the relationship between metal ions and biomineralization, which may become a highly relevant topic in the field.

### 2.5. Magnetic Field-Responsive Hydrogels

Magnetic field-responsive hydrogels are generally composed of matrix hydrogels and magnetic components, which can remotely regulate the physical, biochemical, and mechanical properties due to their structural and functional responses to the external magnetic field. The properties of the magnetic field-responsive hydrogels depend on the composition, concentration, size, and uniformity of the magnetic particles in the hydrogel. When affected by the magnetic field effect, magnetic particles immediately gather, the hydrogel network contracts, and the solvent is “crowded out”, causing the hydrogel shape to change rapidly [75]. Current studies have focused on the application of hydrogels containing magnetic nanoparticles for bone repair. Iqbal et al., synthesized magnetically modified Fe_2_O_3_ nanoparticles (m-nHAP) and added them to a polyvinyl alcohol (PVA) solution to prepare a m-nHAP/PVA hydrogel [76,77]. PVA, with excellent biocompatibility, high mechanical properties, and slow biodegradability, was essential for its application in bone repair. With the increase in m-nHAP content, the pore size inside the hydrogel gradually increased, which facilitated nutrient exchange and significantly increased the adhesion and proliferation of osteoblasts. Mahdavinia et al., recombined chitosan and magnetic Fe_3_O_4_ to produce magnetically responsive gel microspheres for the efficient adsorption of protein [78]. They fixed the Fe_3_O_4_ in situ in an inorganic thickening liquid, mixed the magnetic solution with polyvinyl alcohol and chitosan solution, and then repeatedly froze and melted the mixing solution to obtain the final gel sample. The results showed that, due to the introduction of the magnetic material into the gel microspheres, the maximum adsorption capacity was 240.5 mg/g. Isothermal adsorption data simulation showed that the sample adsorption process was more consistent with the Langmuir model than the Freundlich model. The research group also mixed Fe_3_O_4_ with carrageenan in situ to obtain magnetic nanoparticles, which were then crosslinked with chitosan as a crosslinker [79]. The introduction of magnetic particles into the carrageenan/chitosan complex had significant effects on the multiple properties of the hydrogel, and the results showed that with the increase in magnetic particles, the water absorption and encapsulation rate of the gel to the model drug increased significantly. The combination of xanthan gum and chitosan is also a new exploration. They can self-assemble in the presence of magnetic nanoparticles and form a magnetically responsive polyelectrolyte hydrogel under the action of glucuronic acid [80]. The results showed that the proliferation and adhesion capacity of fibroblasts on the gel were significantly enhanced under the influence of an external magnetic field. The addition of magnetic nanoparticles can also significantly improve the gel’s mechanical strength and improve its rheological energy (increased energy storage modulus). Therefore, it is expected that this magnetic-responsive hydrogel will have potential applications in the field of bone defect repair.

**Figure 3:**
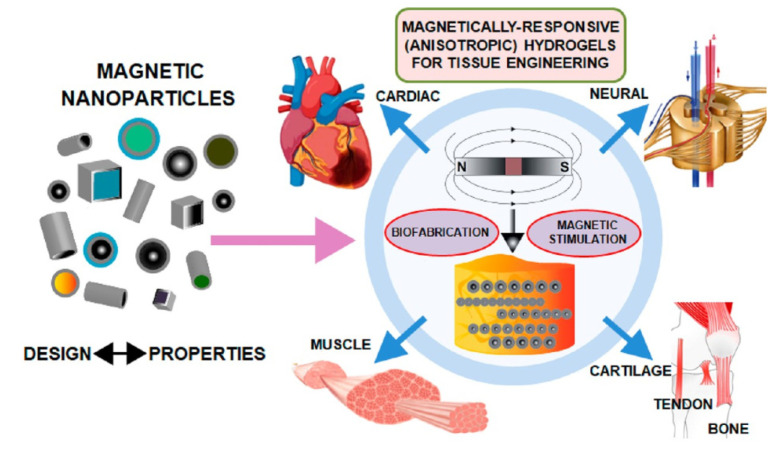
Schematic illustration: use of magnetic hydrogels to engineer different tissues of the human body. Reproduced from [84] with permission. Copyright 2021 American Chemical Society.

It should be mentioned that magnetic materials have been proposed as potential agents to provide hydrogels with the anisotropy required for their use in tissue engineering. The intrinsic properties of magnetic nanoparticles enable their use as magnetomechanic remote actuators to control the behavior of the cells encapsulated within the hydrogels under the application of external magnetic fields. The incorporation of magnetic materials and the subsequent application of magnetic fields may present different advantages for bone tissue engineering strategies. First, magnetic nanoparticles can be used to provide the biomaterials with the visually anisotropic hierarchical architecture in native bone tissues, thus allowing the controlled design of anisotropic magnetically responsive scaffolding materials. In addition, the magnetic forces at the interface between cells and hybrid composites display the capacity for activating the sensitive receptors of the cell’s surface, enhancing cell activity and promoting the bone formation process and the integration of scaffolds into the host bone [81,82,83]. In summary, magnetic hydrogels with anisotropic architectures provide not only an ordered 3D template in which the complex architectural properties of native tissues can be replicated but also add control over cell behavior. Therefore, there are specific strategies in the development of magnetic-responsive hydrogels with the required architectural properties to properly mimic different anisotropic tissues, such as tendons, bone, or cartilage (Figure 3) [84]. However, it has been mentioned that when the magnetic field-responsive hydrogels loaded with concentrated drugs are fixed to a body tissue that is in a pathological state, although the drugs can be accurately released from the hydrogels to achieve targeted controlled release for bone regeneration, the potential toxicity of magnetic nanoparticles derived from the degradation of the hydrogel-based scaffolds should be carefully considered, and the suitable usage concentration, purity, and geometry of the magnetic nanoparticles, as well as the rational fabrication of magnetically responsive hydrogels, remain challenging.

These stimulus-responsive hydrogels can also be used to fabricate constructs that replicate the main physicochemical of other tissues, such as tendons or tendon-to-bone interfaces. For example, Echave et al., developed a gelatin-based multiphasic hydrogel system with a distinct composition and microstructure [85]. In each phase, HAP particles or cellulose nanocrystals (CNC) were incorporated into an enzymatically crosslinked gelatin network to mimic bone or tendon tissue, respectively. Stiffer hydrogels were produced with the incorporation of mineralized particles, and magnetic alignment of CNC resulted in anisotropic structure formation. After the evaluation of biological commitment with human adipose-derived stem cells toward the tendon-to-bone interface, the results revealed aligned cell growth and higher synthesis and deposition of tenascin in the anisotropic phase, which indicated the potential versatility offered by the gelatin–transglutaminase tandem enzyme for the development of strategies to mimic the graded, composite, and complex intersections of the connective tissues.

### 2.6. Enzyme-Responsive Hydrogels

**Figure 4:**
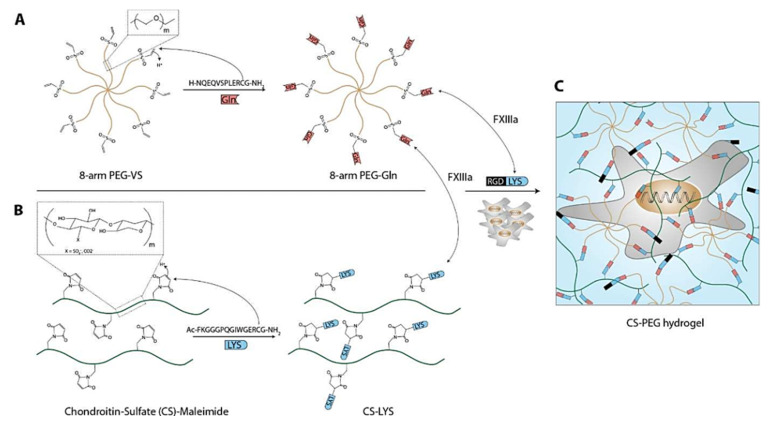
Factor XIII mediated CS-PEG hydrogel formation. The functionalization of (**A**) 8-PEG-VS with TG-Gln peptides resulted in 8-PEG-Gln and (**B**) CS-maleimide with CS-MMP-Lys in CSn-MMP-Lys. (**C**) Formulations of 8-PEG-Gln, CS-MMP-Lys, and the cell adhesion ligand TG-RGD-Lys were crosslinked by FXIIIa in presence of cells to form 3D cellular microenvironments. Reproduced from [89] with permission. Copyright 2016 Elsevier.

Enzyme-responsive hydrogels generally contain enzyme-responsive polypeptides, which can make structural changes in response to specific enzymes, thus promoting the formation or degradation of the hydrogel network. At the design level, enzyme-responsive hydrogels often use natural enzymes present in organisms or abnormally highly expressed in the lesion, such as matrix metalloproteinase (MMP) [86], phosphatase [87], and tyrosinase [88]. In the field of tissue engineering, enzyme-responsive hydrogels, acting as cell and protein carriers, can catalyze the degradation of the hydrogel cytoskeleton, thereby promoting the release of cell growth factors or providing an environment for cell proliferation and differentiation. Anjum et al., prepared a dual-responsive hydrogel based on the ECM sugar 695 aminoglycan (GAG) to regulate the delivery of cell growth factors and stem cell differentiation [89]. Using an MMP-sensitive glutamine transaminase factor XIII (FXIIIa)-specific lysine polypeptide sequence (TG-MMP-Lys) to functionalize the chondroitin sulfate (CS), CS was crosslinked with eight-arm PEG (PEG-Gln) modified by glutamine polypeptide to form a hydrogel. The cell adhesion polypeptide as a model ligand (TG-RGD-Lys) could improve the cell adhesion capacity of the hydrogel. After the encapsulation of bone-forming protein (BMP-2) and BM-MSCs into the hydrogels, BMSCs could maintain cell viability and realize proliferation and migration, and the released BMP-2 could induce the osteogenic differentiation of BMSCs (Figure 4). Therefore, a CS-PEG composite hydrogel can flexibly integrate the molecular tools that are needed to induce various tissues to simulate the characteristics of the extracellular environment, thus achieving the control of cell differentiation and tissue regeneration.

## 3. Conclusions and Perspectives

At present, stimulus-responsive hydrogels are emerging as a new type of smart biomaterial that can sense external physical stimuli (light, temperature, pressure, electric field, magnetic field, etc.), chemical stimuli (pH, redox reaction, ions, etc.), biochemical stimuli (glucose, enzymes, etc.), and other different stimuli. They can respond to stimuli such as the characteristics of the 3D shape and solid–liquid phase state, and exhibit special properties, such as injection ability, self-repair, and shape memory, thus becoming an ideal material to provide tissue engineering scaffold adhesion, cell proliferation, and differentiation, and achieve precise bone defect repair. Nowadays, stimulus-responsive hydrogels not only have the advantages of being highly hydrophilic and good biocompatibility, but also possess regulated stimulus responsiveness, which makes them emerging scaffold materials, widely used for bone defect repair in tissue engineering. However, although many scholars have affirmed the efficacy of bone defect repair through stimulus-responsive hydrogels, their therapeutic effect on large bone defects has not been demonstrated due to the minimally invasive treatment of bone defects, the mechanical strength, and the degradation rate of bone regeneration. Thus, stimulus-responsive hydrogels with biocompatible, osteoconductive, osteoinductive, and osteogenic effects should have mechanical properties matching their degradation rate to meet the complex requirements of large bone defect repair.

During the last few decades, although significant progress ranging from cell biology up to advanced biomaterials has been made for tissue engineering in bone, we still have a long way to go to achieve functional bone tissue with the promise of revolutionizing healthcare by providing artificially engineered functional tissue and organ substitutes. In particular, the following three aspects still need further attention in the future.

First, although hydrogels with biocompatible composition and stimulus response are achievable for tissue-engineered scaffolds, a single response often cannot achieve the ideal treatment effect because of the complexity of the human physiological environment and the diversity of the lesion site environment; thus, a new kind of intelligent nanofiller-loaded hydrogel, such as magnetoelectric nanoparticles with multi-responsive performance, could be combined in the same carrier [90,91,92]. The hydrogel can choose the appropriate means of response according to the characteristics of the environment to achieve the ideal effect.

Second, the extensive development of precise fabrication and personalized medicine treatments for individual complexities will enable the engineering of biomaterials with precise structures and specific functions. Therefore, an in-depth study of the processing technologies and application of additive manufacturing in the biomedical field is needed, owing to its potential to provide personalized solutions for patients. For example, biofabrication is an emerging and rapidly growing research field in which additive manufacturing has been merged with tissue engineering to generate hierarchical tissue-like and personalized constructs. On the basis of 3D bioprinting technology, bioinks combining high-resolution printability with cytocompatibility have been developed as one ideal scaffold material for clinical translation, which will continue to be an active participant in the process of bone regeneration, not only as cells and molecular carriers, but also playing an important role in controlling delivery efficiency and delivery rate, thus making bioprinting hydrogel systems appealing alternatives for tissue engineering and drug delivery purposes, among others [93,94,95,96]. Therefore, it is of great significance to construct osteoblast and osteoclast co-culture models based on 3D printing techniques for promoting bone regeneration and studying the interaction between cells. In addition, the conception of green nanofabrication and the development of microfluidic particles with improved functionalities are essential for the creation of granular hydrogels, which will be also an innovative, green, sustained, and highly promising solution for different therapies in regenerative medicine areas [97].

Finally, further efforts to research hydrogel scaffolds with suitable degradation performance, mechanical properties, and vascular functionalization will be the main aspect and major challenge in the field of bone defect repair—for example, the design and development of extremely sensitive hydrogels that can be manipulated by using extremely weak external stimuli after implantation, thus avoiding the potential toxicity risks associated with some functional but harmful nanofillers (Au NPs, Ag NPs, MNPs) under strong magnetic radiations [88]. Another major challenge is the degree to which biomaterials bind to the local microenvironment in vivo. After biomaterial implantation, the altered microenvironment has a great impact on bone formation, so monitoring the material changes produced in the body in real time is crucial. It is believed that with the continuous progress of bone tissue engineering, stimulus-responsive hydrogels will develop rapidly to provide more solutions for the clinical treatment of bone defects and realize the transformation of clinical results.

## Figures and Tables

**Table 1:** The advantages and limitations of stimulus-responsive hydrogel categories.

Types of Stimuli	Advantages	Limitations
Photo-responsive hydrogels	Mild reaction conditions; Low damage to human body; Spatio-temporal control of drug release without directly contacting the lesion	Ultraviolet and visible light cannot penetrate the tissue, which leads to the limited application only for in vitro system and skin-level treatments
Temperature-responsive hydrogels	injection capacity; Highly targeted and less toxic side effects; Effectively reduce the treatment cost for patients and improve their health-related quality of survival life	Low response rate; Low difference of pathological and normal tissues within the body
pH-responsive hydrogels	The pH of pathological tissues like local tissue inflammation, infection and cancer differs from that of normal tissues	The clinical prediction of the pH value in diseased sites may result in adverse tissue reactions
Redox-responsive hydrogels	Redox-responsive drug release; Relationship between metal ions and mechanical properties; Regulation of hydrogel hardness by redox reaction to promote bone regeneration	Low difference between the pathological and normal tissues limits the application
Magnetic-responsive hydrogels	Directional drug movement in a pathological state under the guidance of the environmental magnetic field can achieve the targeted therapy	The potential toxicity of magnetic nanoparticles may be harmful to live organisms
Enzyme-responsive hydrogels	Structural changes and quick degradation in response to specific enzymes promote the release of bio-factors for cell proliferation and differentiation	Weak peptides activity and low half-life limit the long-term use
